# Ventilation during extracorporeal gas exchange in acute respiratory distress syndrome

**DOI:** 10.1097/MCC.0000000000001125

**Published:** 2024-03-06

**Authors:** Jacopo Fumagalli, Antonio Pesenti

**Affiliations:** a Department of Anesthesia, Critical Care and Emergency, Fondazione Istituto di Ricovero e cura a Carattere Scientifico Ca’ Granda Ospedale Maggiore Policlinico; b Department of Pathophysiology and Transplantation, University of Milan, Milan, Italy

**Keywords:** acute respiratory distress syndrome, extracorporeal carbon dioxide removal, extracorporeal membrane oxygenation, mechanical ventilation

## Abstract

**Purpose of review:**

Accumulating evidence ascribes the benefit of extracorporeal gas exchange, at least in most severe cases, to the provision of a lung healing environment through the mitigation of ventilator-induced lung injury (VILI) risk. In spite of pretty homogeneous criteria for extracorporeal gas exchange application (according to the degree of hypoxemia/hypercapnia), ventilatory management during extracorporeal membrane oxygenation (ECMO)/carbon dioxide removal (ECCO_2_R) varies across centers. Here we summarize the recent evidence regarding the management of mechanical ventilation during extracorporeal gas exchange for respiratory support.

**Recent findings:**

At present, the most common approach to protect the native lung against VILI following ECMO initiation involves lowering tidal volume and driving pressure, making modest reductions in respiratory rate, while typically maintaining positive end-expiratory pressure levels unchanged.

Regarding ECCO_2_R treatment, higher efficiency devices are required in order to reduce significantly respiratory rate and/or tidal volume.

**Summary:**

The best compromise between reduction of native lung ventilatory load, extracorporeal gas exchange efficiency, and strategies to preserve lung aeration deserves further investigation.

## INTRODUCTION

Extracorporeal gas exchange was introduced in critical care to ‘buy time for the lung to heal’ [1]. By preventing hypoxia and hypercapnia, ECMO (Extracorporeal Membrane Oxygenation) could maintain life while relieving the patient's lung from the burden of high pressures and inspired oxygen fractions. In the first randomized clinical trial (RCT) in the field, published in 1979, ECMO was applied in patients with severe acute hypoxemic respiratory failure (AHRF) [2]. Both the ECMO and the control group were ventilated with high tidal volumes (TVs) (10–15 ml/kg), and high pressures (40–50 cmH_2_O plateau pressure); mortality was almost identical (>90%) in both groups, and extracorporeal gas exchange was abandoned except in a few centers [3,4]. Rather unexpectedly, the 2009 Swine influenza H1N1 and the 2019 COVID-19 pandemics led to the explosion of ECMO application worldwide, thanks to the results of the Caesar (2009) and of the EOLIA (2018) trial [5,6]. 

**Box 1:**
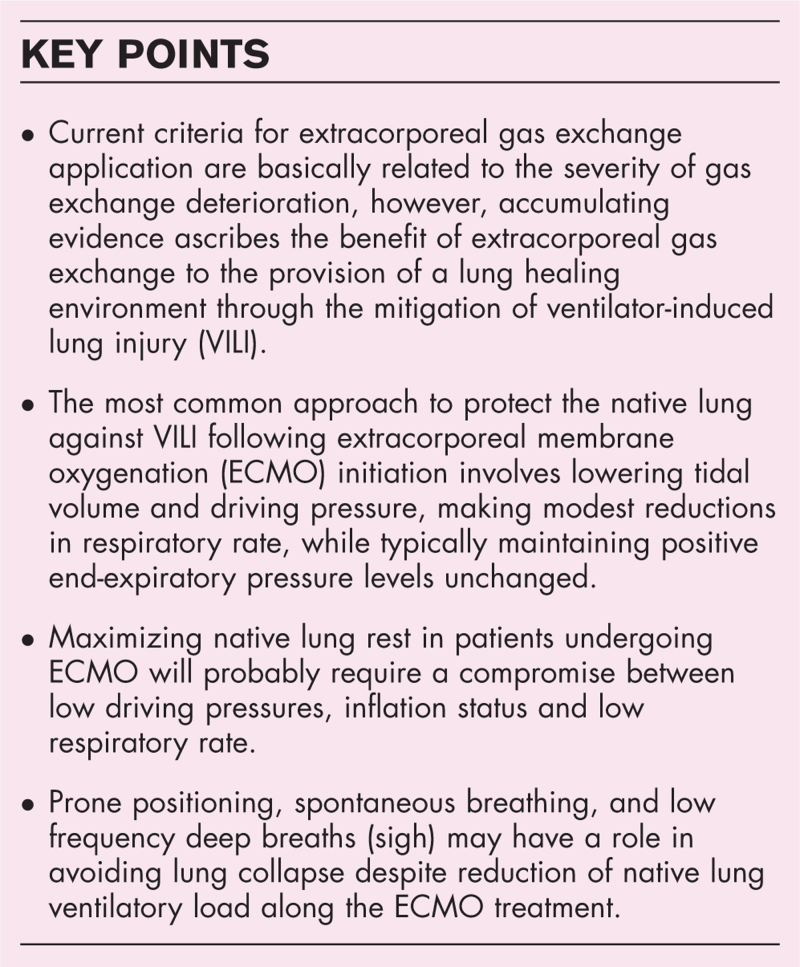
no caption available

Accumulating evidence ascribes the benefit of ECMO to the increased lung protection and VILI reduction. Following the negative results of the first ECMO trial [2], Kolobow and Gattinoni suggested extracorporeal CO_2_ removal (ECCO_2_R) [7], rather than ECMO, as the mean to control the patient's ventilatory needs at much lower blood flows. Although ECMO, which runs at high blood flow (3–5 l/min), provides both oxygenation and CO_2_ removal, ECCO_2_R, which requires much lower blood flows (0.4–2 l/min), is targeted to decrease ventilatory needs in proportion to the amount of CO_2_ removed by the extracorporeal device.

We will discuss the setting of ventilatory support during extracorporeal gas exchange in patients with AHRF. The discussion refers mainly to ECMO, but also to ECCO_2_R when relevant to specific aspects of VILI mitigation. Table 1 summarizes the ventilatory settings before and after extracorporeal gas exchange initiation in the most significant and recent (2019–2023) clinical trials.

**Table 1:** Ventilatory settings before and after extracorporeal gas exchange in most significant & recent (2019–2023) clinical trials

	EOLIA 2018 [6]	LIFEGARDS 2019 [8^▪^]	Chiu *et al.* 2021 [60]	Liao *et al.* 2022 [30^▪▪^]	Lebreton *et al.* 2021 [61^▪^]	Guervilly *et al.* 2022^a^[21^▪▪^]	SUPERNOVA 2019 [17]	REST trial 2021^b^[18^▪▪^]
*Number of patients*	*(n* *=* *124)*	*(n* *=* *350)*	*(n* *=* *152)*	*(n* *=* *62)*	*(n* *=* *302)*	*(n* *=* *18)*	*(n* *=* *95)*	*(n* *=* *202)*
Type of support	ECMO	ECMO	ECMO	ECMO	ECMO	ECMO	ECCO_2_R	ECCO_2_R
Pre-ECMO/ECCO_2_R								
* *Driving pressure, cmH_2_O	18 ± 7	20 ± 7	–	–	18 [14–21]	17 [13–20]	13 ± 4	15 [12–19]
* *Plateau pressure, cmH_2_O	30 ± 5	32 ± 7	–	–	30 [27–3]	31 [25–33]	27 ± 3	26 [23–30]
* *Tidal volume, ml/PBW	6.0 ± 1.3	6.4 ± 2.0	7.7 ± 2.4	6.9 [5.5–9.2]	5.6 [4.9–6.2]	6.5 [5.9–6.9]	6.0 ± 0.2	6.3 [5.8–7.0]
* *Respiratory rate, bpm	30 ± 5	26 ± 8	24 ± 7	21 [16–28]	28 [26–30]	26 [24–30]	27.3 ± 4.8	24 [20–28]
* *PEEP, cmH_2_O	12 ± 4	12 ± 4	12 ± 3	12 [10–15]	12 [10–14]	14 [12–15]	15 [10–16]	10 [8–12]
* *PaCO_2_, mmHg	57 ± 15	68 ± 27	–	38 [33–54]	57 [48–67]	59 [50–71]	48 ± 9	54 [47–63]
* *pH	7.24 ± 0.13	7.24 ± 0.15	–	7.36 [7.25–7.43]	7.31 [7.23–7.37]	7.24 [7.18–7.37]	7.34 ± 0.08	7.30 [7.25–7.37]
* *PaO_2_/FiO_2_, mmHg	73 ± 30	71 ± 34	178 [131–240]	60 [45–73]	61 [54–70]	83 [72–94]	173 ± 61	118 [96–134]
Post ECMO/ECCO_2_R								
* *Driving pressure, cmH_2_O	≈13	14 ± 4	–	–	13 [12–15]	11 [9–15]	9.9 ± 4.3	11 ± 5
* *Plateau pressure, cmH_2_O	≈24	24 ± 7	–	–	25 [24–28]	25 [24–27]	23.5 ± 3.9	26 ± 5
* *Tidal volume, ml/PBW	–	3.7 ± 2.0	6.0 ± 2.2	5.1 [4.0–6.4]	2.9 [2.0–4.4]	3.5 [2.8–4.2]	4.16 ± 0.46	4.5 ± 1.6
* *Respiratory rate, bpm	≈23	14 ± 6	16 ± 4	13 [12–17]	20 [15–22]	15 [11–21]	23.5 ± 6.7	28 ± 6
* *PEEP, cmH_2_O	≈11	11 ± 3	12 ± 3	12 [10–14]	12 [10–14]	14 [12–15]	13.8 ± 3.9	11 ± 3
* *PaCO_2_, mmHg	≈38	42 ± 7	–	31 [27–37]	–	43 [37–51]	46.7 ± 10.4	61 ± 14
* *pH	≈7.37	7.40 ± 0.07	–	7.44 [7.41–7.50]	–	7.40 [7.34–7.45]	7.39 ± 0.08	7.32 ± 0.09

PaCO_2_, arterial partial pressure of carbon dioxide; PaO_2_/FiO_2_, arterial partial pressure of oxygen to fraction of inspired oxygen ratio; PBW, predicted body weight; PEEP, positive end-expiratory pressure.

a Data refers to the control (lung protective) group.

b Data after ECCO_2_R initiation refers to day 3, corresponding to the reported highest average membrane lung CO_2_ clearance.

The LIFEGUARD study, conducted in 23 ECMO centers, shows how the ventilatory parameters applied at ECMO initiation are largely variable between centers [8^▪^]. Accordingly, Marhong *et al.* reported that out of 141 ECMO centers, only 27% declared having a protocol to manage ventilation during VV-ECMO, despite 77% confirmed the goal of obtaining ‘lung rest’ [9]. Obviously, the clinical practice to achieve it are different in different centers.

## TIDAL VOLUME, DRIVING PRESSURE, AND PLATEAU PRESSURE

In the late 1970s, Kolobow *et al.* provided the background on how ECCO_2_R can modulate the ventilatory needs from normal ventilation to very low frequency [respiratory rate (RR) 2–4 bpm] or even complete apnea [10–12]. Low frequency ventilation coupled with ECCO_2_R was applied in patients with severe AHRF, and proved effective in achieving minimal ventilatory load [4,13]. Later the ARMA trial [14] proved that the use of TV of 6 ml/kg Ideal body weight (IBW) improved survival compared to 12 ml/kg. Soon it appeared obvious that if 6 was better than 12 ml/kg IBW, then 4 or 3 ml ml/kg IBW could be better than 6. Terragni *et al.* studied acute respiratory distress syndrome (ARDS) patients ventilated 6 ml/kg (IBW) showing that 30% of them had signs of hyperinflation; when TV was decreased to 4–4.5 ml/kg IBW by low flow extracorporeal CO_2_ removal, hyperinflation decreased and markers of lung protection improved [15]. Various clinical studies from then on reported the use of TVs between 4 and 3 ml/kg IBW, confirming the technical feasibility of the ‘ultra-protective ventilation’ [16,17]. In a larger RCT (REST) the effect of reducing TV to 3 ml/kg IBW by ECCO_2_R was compared to the standard 6 ml/kg IBW [18^▪▪^]. The trial was stopped for futility; no outcome difference was observed between groups. The device in use offered a limited rate of CO_2_ removal (45–85 ml/min), thus forcing the investigators to miss the target 3 ml/kg IBW (the attained average TV in the treatment group was 4.5 ml/kg IBW) and required a slight increase in RR (about 1–2 bpm). Possibly this limited reduction in ventilatory load did not achieve a VILI mitigation sufficient to improve survival. A TV of 3 ml/kg IBW gets very close to ventilating just the anatomical dead space, and therefore requires an almost total metabolic CO_2_ production removal (200–250 ml/min).

At variance with the REST trial, the EOLIA trial used ECMO at blood flows of f 3.5–5 l/min and achieved a decrease of the native lung energy load down from 0.4 to 0.1 J/min/kg; attributable by 60% to reduction in RR and by 40% to reduction in TV [19]. The benefit of reducing TV in ARDS patients is maximized in patients with the lowest compliance (C_RS_), and the highest driving pressure (DP) [20^▪^]. Guervilly *et al.* randomized patients in the early phases of ECMO comparing the EOLIA ventilation strategy (control) with the application of a bundle treatment composed of TV 1–2 ml/kg IBW, RR 5–10 bpm, plus proning and transpulmonary positive end-expiratory pressure [21^▪▪^]. No difference in biochemical markers of biotrauma could be shown. At variance, the reduction of TV was associated to significant decreases in *C*_RS_, leading to DP levels comparable between groups. Reductions in TV can be associated to *C*_RS_ deterioration, with subsequent increase in DP, which might in turn cause further decreases in TV, hindering the expected benefits [22].

Both high driving [23,24] and plateau [25] pressure measured on the first day of ECMO are associated to increased mortality (Table 2). However, the higher driving and plateau pressure suggest a lower *C*_RS_, a proxy of the severity of the disease. Similarly, higher TV and lower driving pressure (likely markers of *C*_RS_ improvement) across the ECMO course have been associated with better outcomes [26] and might be used to determine the timing for ECMO weaning.

**Table 2:** Ventilatory variables associated to survival during extracorporeal gas exchange

Ventilation parameter	Association with outcomes	References
* Mechanical power*		
	Higher mechanical power levels along first 3 ECMO days associated to worse outcome	[60]
* PEEP*		
	Lower PEEP during first 3 days of ECMO independently associated with higher mortality	[62]
* Driving pressure*		
	Higher dynamic DP independently associated with worse outcomes	[24]
	Higher DP is independently associated with higher in-hospital mortality	[23]
	Early DP reduction after ECMO initiation for ARDS predict negative outcomes	[26]
* Plateau pressure*		
	Higher plateau pressure on the 1st ECMO day associated to increased mortality	[25]
* Respiratory rate*		
	Higher respiratory rate during first 3 ECMO days independently associated to reduced survival	[30^▪▪^]
* Prone positioning*		
	Prone positioning (within 5 days from ECMO initiation) improves survival	[51]

ARDS, acute respiratory distress syndrome; DP, driving pressure; ECMO, extracorporeal membrane oxygenation.

We should also take into consideration that low constant TV ventilation causes surfactant dysfunction, increasing alveolar surface tension and favoring lung collapse. This effect might be avoided by the use of a sigh at predefined intervals [27^▪^]. In a recent RCT the application of a sigh (peak pressure 30 cmH_2_O for 3 s) once per minute in patients with AHRF undergoing pressure support ventilation, was well tolerated and increased oxygenation while reducing TV and RR [28^▪^]. Of note, in a seminal experience of low frequency ventilation during ECCO_2_R, patients were managed with a RR of 2–4 bpm reaching up to 35 cmH_2_O (inspiratory time 2 s) on an average positive end expiratory pressure (PEEP) of 15.4 ± 3.3 cmH_2_O [4]. Although there was no observed decrease in *C*_RS_, drawing any conclusions regarding the impact of this ventilatory strategy on patient outcomes is not warranted.

## RESPIRATORY RATE AND EXPIRATORY TIME

A wide range of frequency, from 4 to 30 breaths per minute has been reported: however, in most lung protective ventilation studies RR has been increased whenever needed to counteract the PaCO_2_ increase due to reduced TV. Despite the concept of mechanical power has been widely recognized as a robust model to quantify the amount of potential damaging energy transferred to the lung, criticism has been raised regarding the contribution of the individual mechanical power determinants in generating VILI [29]. Higher RR appears to contribute to VILI and worsened outcomes in ARDS patients: a recent analysis however, demonstrated that, per unit change, DP (cmH_2_O) was four times more powerful than RR (bpm) in increasing mortality rate [30^▪▪^]. Higher RR during ECMO has been independently associated to increased mortality rate [31] (Table 2). Robust preclinical evidence demonstrated the beneficial effects of reducing RR in animal models of ARDS [32–35]. In a severe ARDS model caused by injurious ventilation and supported with ECMO, the early fibroproliferative response was better prevented by applying near-apneic ventilation (RR at 5 bpm) than by conventional protective ventilation. However, *C*_RS_ decreased over time during near apneic ventilation (5 bpm) with 10 cmH_2_O DP and 10 cmH_2_O PEEP [36^▪▪^]. Indeed, an increase in expiratory time, often associated to the decrease in rate, may cause alveolar collapse [37]. At variance, a short expiratory time may reduce cyclic recruitment and derecruitment, with better distribution of ventilation [38]. Thus, the interaction between RR, size of TV and PEEP requires further experimental and clinical evaluation to maximize its possible beneficial effects on outcome.

## POSITIVE END EXPIRATORY PRESSURE

Since the 1979 ECMO RCT [2], many ECMO studies have reported a progressive decrease of C_RS_ after ECMO initiation, most likely related to lung collapse [39] favored by lowering TV and RR. Accordingly, radiologic confirmation of worsening lung imaging has been documented after ECMO initiation [40^▪^]. In order to prevent compliance decay when reducing TV, a higher PEEP may be effective [41]. Indeed, it was shown that a PEEP >20 cmH_2_O was necessary to prevent lung collapse in apneic lambs with healthy lungs [11].

According to the available data, though, the decrease in *C*_RS_ observed after ECMO/ECCO_2_R initiation in general does not seem to call for a PEEP higher than 10 cmH_2_O [8^▪^]. At variance Marhong *et al.* reported a different management in ECCO_2_R studies where PEEP was increased from 13>17 cmH_2_O after extracorporeal support initiation [42]. Brusatori *et al.* targeted a constant mean airway pressure before and after extracorporeal gas exchange support by PEEP increase. The modest PEEP change (+1–2 cmH_2_O) could not prevent the loss of *C*_RS_ due to the reduction of both TV and RR [43^▪^].

As in most patients with ARDS physiological phenotyping has been shown to be clinically useful. The assessment of lung recruitment, either by imaging [44] or by lung mechanics,(might help identifying the PEEP level guaranteeing alveolar aeration while avoiding overdistension [45]. Bedside EIT monitoring at ECMO initiation might further help by monitoring the end-expiratory lung volume trend and the distribution of ventilation [46].

Lastly, Wang *et al.* in a single center RCT tested the efficacy of setting PEEP in order to obtain a positive end expiratory transpulmonary pressure in AHRF patients requiring ECMO support [47]. The authors detected, in the transpulmonary pressure guided PEEP group, a significantly higher proportion of patients weaned from ECMO, shorter duration of ECMO support and reduced mortality rate at 60 days. Caution should be used in interpreting these results since the transpulmonary pressure guided PEEP group received also lower VT and DP compared to the control group.

## MODE OF MECHANICAL VENTILATION

Most patients, at the time of extracorporeal gas exchange initiation, receive intermittent mandatory ventilation. A worldwide survey in 144 ECMO centers reported pressure control as the most frequently used (64%) mode of mandatory ventilation [48]. This is likely aimed to a close control of the driving pressure and the achievement of a higher mean airways pressure level (possibly improving oxygenation). However, potential drawbacks of a PCV approach include: higher inspiratory flow, which is associated with increased markers of VILI in ARDS patients [35]; compared to volume control, PCV requires a higher PEEP level to prevent C_RS_ loss, particularly when low TV are used [49].

Some authors are proposing a paradigm shift from protective lung ventilation (decreasing TV) and/or open lung approach (minimizing driving pressure and raising PEEP) to a ‘time controlled adaptive ventilation’ exploiting the features of airways pressure release ventilation (APRV). This mode takes advantage of the mechanical characteristics of the diseased lungs providing prolonged (4–6 s) CPAP time in order to allow recruitable lung opening while avoiding unstable alveoli closure by setting a very short expiratory time [50]. A warning might be put forward since this approach can result in substantially higher mean airways pressures, similar to high frequency ventilation [51], possibly interfering with the hemodynamics.

## PRONE POSITIONING

Despite proning improves survival in patients with moderate to severe ARDS [52], the LIFEGUARD study reported that only 15% out of 350 patients were proned at least once during ECMO [8^▪^]. Such a scanty use is probably caused by fear of possible complications such as cannula displacement, bleeding, or other mechanical complications No major prospective RCT is available to evaluate proning in patients undergoing ECMO; however, a recent review and meta-analysis concluded that proning in ECMO improves outcome. Early proning (within 5 days from connection) appears an important determinant of a significant survival advantage possibly associated to an improvement in *C*_RS_[53^▪^].

## ASSISTED/SPONTANEOUS BREATHING

While awake nonintubated ECMO /ECCO_2_R contributes to avoid muscle deconditioning in patients with chronic pulmonary disease or awaiting lung transplantation, awake ECMO has been much less common in AHRF patients. A recent review of the literature identified 467 adults undergoing awake ECMO for AHRF. Failure (need for intubation) was reported in 34% of cases [54].

In AHRF patients, assisted breathing can result in multiple physiological benefits [55]: however, excessive patients’ respiratory effort carries the risk of patient self-inflicted lung injury [56]. The Karolinska group reported a very low mortality rate (24*%)* in 17 severe ARDS patients treated with extracorporeal support coupled with minimal sedation and pressure support ventilation; the authors accepted arterial oxygenation values lower than those before connection to ECMO [57].

Spinelli *et al.* analyzed the spontaneous breathing pattern during maximum extracorporeal CO_2_ removal in 15 ARDS patients undergoing ECMO since less than 3 days [58^▪^]. Sixty percent of patients achieved a physiological breathing pattern when the ECMO sweep gas flow was increased to achieve clearance of the entire patients’ CO_2_ production. Patients with higher SOFA score, very high CO_2_ production, and disease severity at the CT scan showed a rapid shallow breathing pattern even at maximal ECMO CO_2_ removal.

In the course of ECMO the improvement in the patient's conditions can include the recovery of the control by blood gases of respiratory drive, largely overruled in the most severe phases by other triggers, leading to high rate shallow breathing, even in presence of normal arterial blood gases and pH [59]. Monitoring the patient's response to changes in the rate of CO_2_ removal may predict the feasibility of assisted breathing [60]: to exploit the advantages of assisted breathing during ECMO, both adequate patient selection (i.e., Identifying those responsive to titration of sweep gas flow with changes in drive and effort), and monitoring of patients drive and effort is recommended.

## CONCLUSION

When ECMO was in its infancy, the recommended duration of ECMO in absence of signs of recovery was measured in days. Today multiple reports of prolonged ECMO courses (quite a few lasting more than 100 days) describe the possible late recovery of lung function [61^▪^]. This is undoubtedly linked to technology improvements, but also to confidence in the healing effect of a protective lung environment.

At the present time the accepted criteria for ECMO application are basically related to severe gas exchange deterioration. ECMO however, though ensuring safe blood gases, is further justified when it provides conditions that favor lung healing by minimizing ventilator induced lung injury.

The benefit will be maximized for patients in whom mechanical ventilation is maintaining life at a very high cost in terms of VILI and/or hemodynamic impairment (right ventricular dysfunction, high central venous pressure). We can foresee a future in which the risk justifying ECMO application will not be measured just in terms of gas exchange impairment, but more explicitly in terms of VILI potential and systemic adverse effects, accepting at times even a less severe gas exchange impairment.

A better ventilatory management of patients undergoing ECMO will probably come from a compromise between low driving pressures, inflation status and low RR. In this compromise the role of sigh, a very low frequency deep breath (pressure limited) may regain importance after years of oblivion [62,63^▪^,^64^].

## Acknowledgements

*None.*

### Financial support and sponsorship

*None.*

### Conflicts of interest

*J.F. does not have any conflict of interest. A.P. reports personal fees from Baxter, Maquet, Boehringer Ingelheim, Xenios outside the submitted work.*

## REFERENCES AND RECOMMENDED READING

Papers of particular interest, published within the annual period of review, have been highlighted as:

▪ of special interest
▪▪ of outstanding interest
